# Supplementary figures and images for: Elucidating the Associated Biological Function and Clinical Significance of RHOJ Expression in Urothelial Carcinoma
Source: Int J Mol Sci. 2023 Sep 14;24(18):14081. doi: 10.3390/ijms241814081 (PMC10531362; doi:10.3390/ijms241814081)

GSE32894  
Imsig signature

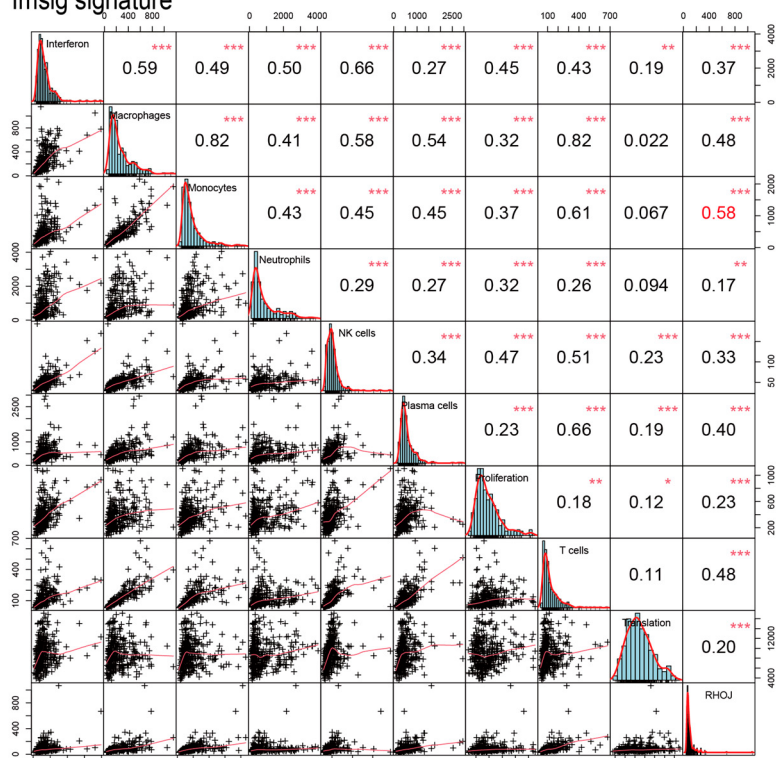

TIDE signature

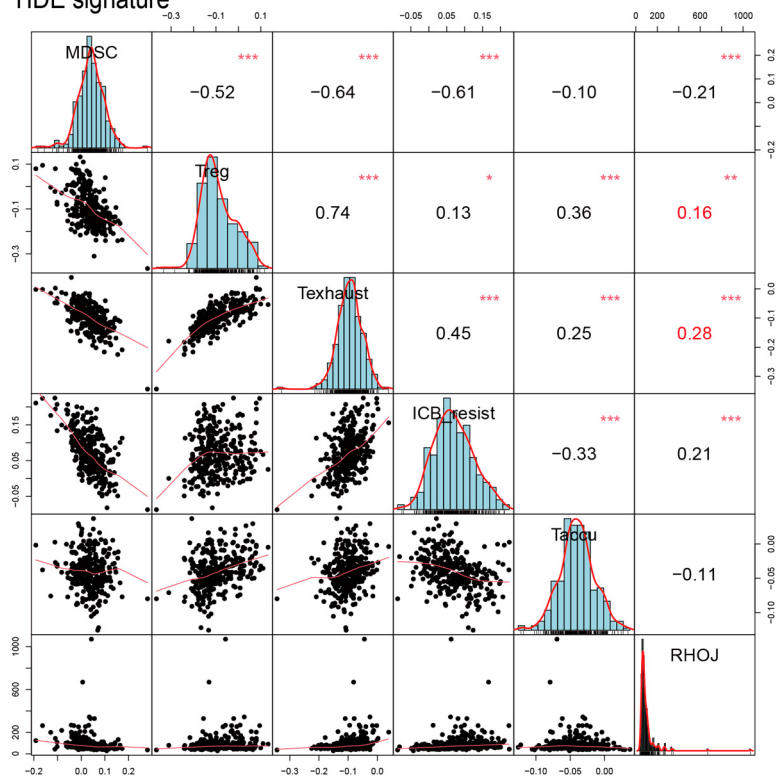

Figure S1: RHOJ correlation to ImSig and TIDE in GSE32894. \*  $p < 0.05$ , \*\*  $p < 0.01$ , \*\*\*  $p < 0.001$ .

Supplement: Supplementary file 1 [file ijms-24-14081-s001.zip › Supplementary figure S1.pdf]
